# Editorial: Reviews and applications of implementation research in aging and public health

**DOI:** 10.3389/fpubh.2026.1783901

**Published:** 2026-02-16

**Authors:** Shinduk Lee, Matthew Lee Smith, Marcia G. Ory

**Affiliations:** 1 College of Nursing, University of Utah, Salt Lake City, UT, United States; 2 School of Public Health, College Station, Texas A&M University, TX, United States; 3 Center for Community Health and Aging, College Station, Texas A&M University, TX, United States

**Keywords:** aging, implementation determinants, implementation framework, implementation science, implementation strategies, public health

## Abstract

Implementation research plays a vital role in translating evidence-based interventions into real-world settings and addressing the unique challenges faced by aging populations. This editorial provides an overview of a Research Topic containing 21 manuscripts aimed at fostering evidence-based practices in the field of implementation research and exploring strategies to improve the delivery and effectiveness of interventions for older adults. The articles within the Research Topic centered around four themes: (1) application of implementation theories, models, and frameworks; (2) implementation strategies; (3) evaluation of interventions and their implementation; and (4) cross-cutting issues. Collectively, this collection of manuscripts underscores the importance of integrating dissemination and implementation considerations across all stages of an intervention, from its development through implementation and evaluation.

## Background

A major demographic shift in the 21st century is underway. By the late 2070s, the number of adults aged 65 years and older is projected to reach 2.2 billion, surpassing the number of children (1). This demographic transition represents an achievement, which will bring with it unique challenges and opportunities from an aging and public health perspective. Older age is associated with increased prevalence of chronic conditions and geriatric syndromes such as physical, cognitive, and social frailty. While these trends pose clear challenges, they also offer an opportunity to promote healthy aging and advance health equity among older adults.

A wide range of interventions such as evidence-based programs, clinical practices, policies, or other structured activities, have been developed to improve health outcomes for older adults and support system-level improvements. While many have shown efficacy in research settings, difficulties persist when translating the evidence into meaningful public health impact. Implementation research plays a vital role in this translation by identifying the factors and mechanisms that influence how interventions are implemented, adopted, scaled, and sustained in community and clinical settings. Further, implementation research is integral when testing and adapting strategies to assess their feasibility and integrate interventions into real-world practice.

This Research Topic, “*Reviews and Applications of Implementation Research in Aging and Public Health*,” features a curated collection of 16 review articles, along with single-study articles and perspective papers addressing various implementation research perspectives. The 21 articles cover a wide range of topics—from health behaviors of older adults to hidden workers in aging populations. The contribution also represented geographic diversity, including research from Asia (Chen et al.; Lv et al.), Australia (Clemson et al.; Nagar et al.), North America (Martin et al.; Paone et al.; Zhu et al.), and low- and middle-income countries (Mosha and Ngulube), along with reviews that synthesized evidence across multiple regions (Estabrooks et al.; Ferrero-Sereno et al.; Lee and Kang; Li, Wang, Ren et al.; Li, Wang, Li et al.; Nsobundu et al.; Shi et al.; Tian et al.; Yang et al.; Yao et al.). By bridging diverse fields and geographic contexts, this Research Topic facilitates the exchange of insights among researchers and strengthens the collective understanding of implementation in aging and public health.

Several sub-topics emerged from the papers in this Research Topic, which include: (1) application of implementation theories, models, and frameworks; (2) implementation strategies; (3) evaluation of interventions and their implementation; and (4) cross-cutting issues.

## Application of implementation frameworks

The expanding use of implementation theories, models, and frameworks reflects substantial progress in the field by providing a structured approach to translating research into practice, understanding the process of implementation, and evaluating implementation outcomes. This sub-topic highlights how these frameworks are being applied within aging and public health in three articles.

Chronic diseases are prevalent within the older adult population with diabetes being seen as a global public health problem (2). Diabetes self-management programs (DSMPs) are strongly recommended to equip individuals with the knowledge, skills, and support needed to improve diabetes-related health outcomes and prevent or delay diabetes-related health complications. Nsobundu et al. applied the RE-AIM and PRISM (Practical, Robust Implementation and Sustainability Model) frameworks to assess the process evaluation outcomes of traditional, group-based DSMPs. The review found uneven reporting across RE-AIM/PRISM domains, with adoption, implementation, maintenance, and contextual factors reported far less frequently than patient-level outcomes such as reach and effectiveness. This gap underscores the need for standardized reporting across different domains to better assess and strengthen the scalability and sustainability of DSMPs.

A major aging and public health implementation issue is how organizations can scale and sustain their health promotion and implementation programs. Paone et al. addressed this question in their examination of Community Aging in Place—Advancing Better Living for Elders (CAPABLE) by organizing their research efforts around the RE-AIM (Reach, Effectiveness, Adoption, Implementation, and Maintenance) and CFIR (Consolidated Framework for Implementation Research) frameworks. Analyzing a range of primary and secondary data from home care visits that combined clinical care and home modifications, the authors examined implementation outcomes and influencing factors that enable older adults with limitations in daily activities to age-in-place. This paper demonstrates the practical utility of established implementation science frameworks, setting an excellent example of how diverse data sources can be leveraged for systematic assessment of program implementation.

Older adults, who are disproportionately affected by complex health needs (3), encounter challenges related to fragmented health systems, polypharmacy, and the safe use of medications. Changaris applied CFIR to design a team-based primary care model that emphasizes medication safety, efficacy, and adherence (SEA). The framework guided the integration of implementation strategies and tools, strengthening the SEA model's practicality. Whereas, Paone et al. and Nsobundu et al. used implementation frameworks primarily to evaluate implementation and identify influencing factors, the paper by Changaris shows a different use of such frameworks by applying one to guide the development of a primary care model aimed at improving care outcomes.

## Implementation strategies

Implementation strategies can be broadly defined as methods or techniques to improve implementation outcomes (4). This sub-topic includes 4 papers that collectively advance understanding about implementation strategies through their respective emphases on identification, development, or synthesis.

Participant recruitment has been a consistent challenge reported across a wide range of interventions, including those focused on health promotion, disease prevention, and self-management. Estabrooks et al. summarized recruitment strategies used to deliver lifestyle interventions in community and clinical settings, along with the reporting of these strategies and their associated reach outcomes. The scoping review found place-based strategies to be most effective and that lower recruitment rates were observed among older adults compared with younger age groups. This could indicate a unique context of reaching different age groups, underscoring the importance of considering intended populations and settings when planning recruitment strategies.

Information and communication technology (ICT) can enhance the delivery of health programs by reducing geographical barriers and improving accessibility, reach, and efficiency. Two systematic reviews by Li, Wang, Ren et al. and Tian et al. examined ICT-based health programs. Li, Wang Ren et al. focused on ICT-supported Tai Chi interventions for individuals with mild cognitive impairment and reported improvements in cognitive and physical performance. Tian et al. found that ICT-based integrated care improved quality of care, although evidence on cost-effectiveness was mixed. They proposed several possible explanations, including intervention duration, participant characteristics, health outcome improvements, and the degree of proactive implementation. These findings highlight the need for more systematic documentation of implementation contexts and strategies to better assess the cost-effectiveness of ICT as an implementation approach and to support scalability and sustainability.

The core idea of humanistic care emphasizes person-centered approaches that integrate clinical excellence with compassion, empathy, and respect for patients and providers. However, its implementation often remains predominantly patient-focused and faces numerous barriers, including limited provider training in humanistic care (e.g., relational skills), challenging workplace conditions (e.g., high turnover rates), and influential inner and outer settings (e.g., leadership support and related policy) (5). Addressing these barriers, Lv et al. proposed a dual-subject model of humanistic care that is provider-centered and patient-oriented. A key innovation of this model is its strong focus on provider-centered strategies, which include establishing a humanistic care management team, implementing various activities to cultivate humanistic competencies among providers, and fostering a humanistic environment within the hospital. The model improved providers' capacity to deliver humanistic care and enhanced satisfaction among patients and providers.

## Evaluation of interventions and their implementation

Efficacy and effectiveness have been among the most extensively examined aspects of intervention delivery, likely because understanding their impact is essential for justifying implementation efforts. This sub-theme includes eight papers that address intervention effectiveness or efficacy, as well as those reporting other implementation outcomes and factors influencing implementation.

Three review articles examining outcomes of physical activity interventions were included in the Research Topic. A network meta-analysis by Li, Wang, Li et al. examined the effectiveness of dance interventions for reducing fall risks among older adults and demonstrated meta-analytic evidence that dance interventions positively impact balance and mobility. Two meta-analyses by Chen et al. and Yang et al., respectively, summarized the efficacy of Ba Duan Jin (a traditional Chinese exercise also known as the Eight-Section Brocade), each centering on older adults but with a different focus. Chen et al. examined its role in cardiovascular disease prevention, while Yang et al. evaluated its effects on balance for fall prevention. Both reviews reported improvements in their respective outcomes. Notably, Yang et al. identified optimal dosing parameters for the intervention's balance outcomes—an essential piece of information that strengthens the interpretability of intervention effects, supports replicability, and informs decisions about scaling and adaptation.

Other contributions in the Research Topic explore themes beyond physical activity, including mind–body therapies, technology-based caregiver interventions, and primary care or pharmacological approaches. The scoping review by Martin et al. synthesized evidence on the feasibility, acceptability, and cardiometabolic risk–reduction effects of mind–body therapy interventions among Black Americans. Shi et al. evaluated the effectiveness of meditation, a type of mind-body therapy intervention, in older adults with subjective cognitive decline, cognitive impairment, and Alzheimer's disease. Ferrero-Sereno et al. presented a systematic literature review, which found that technology-based interventions were generally effective in improving health-related quality of life, emotional distress, caregiver burden, and perceived social support among caregivers of people with dementia. Nagar et al. presented a review of seven Australian guidelines on the pharmacological management in dementia care and identify patient-, provider-, and system/support-related barriers preventing effective implementation of the guidelines.

It has long been recognized that translating research into practice involves considerable delays and difficulties in implementing interventions in real-world settings. Effectiveness–implementation hybrid designs were introduced to help address these challenges (6). Clemson et al. described a pragmatic hybrid type 2 mixed-methods implementation and effectiveness study to engage general medical practitioners in fall prevention. Examining often neglected setting-level implementation outcomes, this paper offers a strong example of an effectiveness–implementation hybrid design and the implementation of evidence-based approaches, incorporating a “reflection and expansion” process to enhance sustainment and promote broader dissemination.

## Cross-cutting issues

Offering broader perspectives on aging and public health, this sub-topic includes a set of 6 diverse contributions that address other important aspects of aging and public health from varying angles.

A perspective paper by Bacsu et al. shared insights about the importance of publishing review protocols to support comprehensive search methodology, allow reviewer feedback to improve search methodology, and reduce risk of unintended research replication. The authors also proposed three strategies to advance review protocols in implementation science. Bannon et al. identified single-session interventions as a potential intervention to meet unmet mental health needs among older adults and emphasize the role of implementation science frameworks (e.g., CFIR, RE-AIM) in supporting effective implementation. In their meta-narrative review, Lee and Kang introduced a framework of transdisciplinary research for older hidden workers, providing potential guidance for the intervention design, implementation, and evaluation for this population. Yao et al., Mosha and Ngulube, and Zhu et al. contributed broader perspectives through reviews on multimorbidity assessment tools, data-sharing barriers for chronic disease prevention in low- and middle-income countries, and healthcare access barriers among older Chinese immigrants in Canada.

## Conclusions

The diversity of topics represented in this Research Topic reflects the expansion of implementation science within aging and public health research. Notably, the papers collectively underscore the importance of integrating dissemination and implementation considerations across all stages of an intervention, from its development through implementation and evaluation. It has been over 25 years since the introduction of the RE-AIM framework (7, 8), now one of the most widely used implementation frameworks, and more than a decade since Nilsen's seminal paper outlining implementation theories, models, and frameworks (9). These milestones reflect substantial progress in the field, and a greater appreciation of the importance of context for understanding how best to scale and sustain evidence-based practices, programs, and policies that can promote healthy aging at every age for everyone. Although persistent research gaps remain in terminology, measurement, and reporting of intervention and implementation strategies, papers in this Research Topic demonstrate the successful application of implementation frameworks and make a strong case for more proactive integration of implementation theories, models, and approaches in aging and public health. Together, these contributions point to a field that continues to evolve and mature.
